# ADHD: Development of a printable poster for parents, teachers and healthcare professionals

**DOI:** 10.1192/j.eurpsy.2024.933

**Published:** 2024-08-27

**Authors:** I. Belabbes

**Affiliations:** hospital arazi, sale, Morocco

## Abstract

**Introduction:**

ADHD is a very frequent reason for consultation in child psychiatry. It affects 2.5% of children and 5% of adults. Diagnosis is clinical. Treatment is based on medication combined with psychosocial interventions.

**Objectives:**

Develop an ADHD guide for caregivers.

**Methods:**

We carried out a literature review covering the last 5 years using the google scholar and pubmed search engines, including the key words ADHD, in order to produce a printable guide for caregivers working with children, in particular school teachers and healthcare personnel.

**Results:**

Attention deficit hyperactivity disorder (ADHD) is a neurodevelopmental disorder affecting 5% of school-age children.

It is characterized by abnormally high levels of developmental inattention, hyperactivity and impulsivity, leading to impaired personal, social, academic or occupational functioning. Because of its pervasiveness, ADHD can interfere negatively with general well-being, as well as with social life, academic performance and the development of social skills, which can lead to low self-esteem.

ADHD has multiple etiologies. It is thought to be due to a complex interaction between genes and environment. In fact, genetic vulnerability predisposes to the disorder which, under the influence of an unfavorable environment, expresses itself in clinical symptoms represented by 2 dimensions: inattention, hyperactivity and impulsivity.

Diagnosis is essentially clinical, and treatment is based on medication combined with psychosocial interventions.

**Conclusions:**

ADHD is one of the most frequently encountered disorders in general practice, pediatrics and child psychiatry. Early recognition of the disorder enables appropriate management, while limiting the impact of the disease on the functioning of the young person and his or her family.

**Disclosure of Interest:**

None Declared

